# Milano–Torino Staging and Long-Term Survival in Chinese Patients with Amyotrophic Lateral Sclerosis

**DOI:** 10.3390/cells10051220

**Published:** 2021-05-17

**Authors:** Ruojie He, Minying Zheng, Ling Lian, Xiaoli Yao

**Affiliations:** Guangdong Provincial Key Laboratory of Diagnosis and Treatment of Major Neurological Diseases, National Key Clinical Department and Key Discipline of Neurology, Department of Neurology, The First Affiliated Hospital, Sun Yat-sen University, No. 58 Zhongshan Road 2, Guangzhou 510080, China; herj@mail2.sysu.edu.cn (R.H.); zhengmy@mail.sysu.edu.cn (M.Z.); lianling@mail.sysu.edu.cn (L.L.)

**Keywords:** amyotrophic lateral sclerosis, Milano–Torino staging, long-term survival, neuropsychiatric factors

## Abstract

(1) Background: The aim of this longitudinal study was to evaluate the association between disease progression according to the Milano–Torino staging (MITOS) system and long-term survival in Chinese patients with amyotrophic lateral sclerosis (ALS). We also examined factors affecting MITOS progression. (2) Methods: Patients were enrolled and underwent follow-up at 6, 12, 18, and 24 months, and their demographic and clinical data, including the Milano–Torino stage, Amyotrophic Lateral Sclerosis Functional Rating Scale—Revised (ALSFRS-R) score and neuropsychiatric data, were evaluated. The sensitivity and specificity of predicting survival outcomes based on MITOS progression and ALSFRS-R score decline from baseline to 6 months were compared. The associations between MITOS progression from baseline to 6 months and survival outcome at 12, 18 and 24 months were examined, and factors associated with disease progression were evaluated with subgroup analyses. (3) Results: Among the 100 patients included, 74% were in stage 0 at baseline, and approximately 95% progressed to a higher stage of the MITOS system at 24 months. MITOS progression from baseline to 6 months and ALSFRS-R decline showed comparable value for predicting survival at 12, 18, and 24 months. MITOS progression from baseline to 6 months is strongly associated with death outcomes. Older age at onset and increased depression and anxiety scores may be related to disease progression. (4) Conclusions: MITOS progression during the early disease course could serve as a prognostic marker of long-term survival and may have utility in clinical trials. Age at onset and diagnosis and neuropsychiatric factors might be associated with disease progression.

## 1. Introduction

Amyotrophic lateral sclerosis (ALS) is a lethal neurodegenerative disease that affects both upper and lower motor neurons, resulting in progressive spinal and bulbar paralysis as well as respiratory failure, with a survival time of 3–5 years. Patients with ALS have a high degree of heterogeneity in phenotype, and genetic, environmental and clinical factors are associated with survival, making prognostic evaluations challenging [1,2]. The survival time is a widely used primary outcome in clinical trials, though a considerable amount of time and money are needed, making them ineffective and lengthy. Reliable and early biomarkers for disease progression and prognosis prediction would help shorten the duration of clinical trials and facilitate precise treatment for ALS. The Amyotrophic Lateral Sclerosis Functional Rating Scale—Revised (ALSFRS-R) score is based on impairment in four functional domains: the bulbar, fine motor, gross motor, and respiratory domains. It is widely used in clinical trials to evaluate functional decline and survival [3,4,5]. However, the ALSFRS-R score is multidimensional and lacks milestones, and as a single metric, it has a floor effect, which hinders its application in measuring functional deterioration in the late stage of diseases in clinical trials. Some clinical trials have reported that negative findings were obtained partially due to the employment of ALSFRS-R decline as the primary endpoint [6,7].

At present, two ALS staging systems, the Milano–Torino staging (MITOS) system and the King’s College staging system, have been proposed to satisfy the urgent need for disease course measurement [8,9]. The MITOS system is based on loss of function in four key domains (walking or self-care, swallowing, communicating, and breathing) in the ALSFRS-R scale. Briefly, stage 0 is defined as no loss of function in any domain, stages 1–4 are defined as loss of function in one to four domains, and stage 5 is defined as death [8,10]. The King’s system, based on the involvement of three anatomical regions (bulbar, upper limb, and lower limb), includes five stages (stages 1–3 are defined as the involvement of one to three regions, stage 4 is defined as requiring gastrostomy or noninvasive ventilation, and stage 5 is defined as death) [9,11]. Both staging systems have been evaluated in several population-based studies [12,13]. Tramacere et al. performed a randomized controlled trial (RCT) in Italy and validated the ability of the MITOS system to measure disease progression and showed that progression in the first 6 months based on the MITOS system could reliably predict the prognosis of ALS for up to 18 months [10]. Furthermore, a multicenter study from Europe developed and validated a model incorporating age at onset, site of onset, ALSFRS-R rate of progression, delayed time to diagnosis, forced vital capacity (FVC) and cognitive loss to predict a composite survival endpoint in patients with ALS [14]. Since the genetic background and lifestyle of Chinese patients are different from other patients, the direct use of results from other countries may lead to incorrect assessments. It is important to re-explore prognostic markers and validate the value of the MITOS system in predicting long-term survival outcomes in Chinese patients with ALS.

In the present study, we aimed to evaluate the association of progression according to the MITOS system during the early disease course and long-term survival outcomes (up to 24 months) in Chinese patients with ALS. Moreover, we explored the demographic and clinical factors related to disease progression measured with the MITOS system.

## 2. Methods

### 2.1. Patients and Study Design

From December 2015 to September 2017, patients who met the inclusion criteria were enrolled in the registry at the Department of Neurology, First Affiliated Hospital of Sun Yat-sen University. Inclusion criteria were as follows: patients aged 18–75 years; diagnosed with definite, probable or probable laboratory-supported ALS according to the El Escorial revised criteria; and FVC ≥ 70% of the predicted value at the baseline visit. The primary outcome was death. At the baseline assessment, patient demographics and clinical variables, including sex, age at onset, age at diagnosis, delayed time to diagnosis, region of onset, and total ALSFRS-R score, were collected. Patients were evaluated at study entry and at 6-month, 12-month, 18-month, and 24-month follow-up visits, and information including stage and progression based on the MITOS system, ALSFRS-R decline, and neuropsychiatric assessment scores was recorded. This study was approved by the Ethics Committee of The First Affiliated Hospital, Sun Yat-sen University. All participants gave their informed written consent prior to the study.

### 2.2. The MITOS System in ALS

The MITOS system was based on the loss of independence in four key domains: walking or self-care, swallowing, communicating, and breathing. Each functional item had a threshold score that indicated loss of function in the specific domain. Subscores for each item that was below the threshold were assigned a functional score of 1, while a functional score of 0 was assigned when the subscores were above the threshold. The stages were determined by the sum of these functional scores across the four key domains, and the five stages of the MITOS system were defined as follows: functional involvement but no loss of function in any domain was defined as stage 0; loss of function in one domain was defined as stage 1; loss of function in two domains was defined as stage 2; loss of function in three domains was defined as stage 3; loss of function in four domains was defined as stage 4; and death was defined as stage 5.

### 2.3. Anxiety, Depression, and Sleep Quality Assessments

Anxiety and depression were diagnosed in ALS patients using the Hamilton Anxiety Scale (HAMA) and Hamilton Depression Scale (HAMD), respectively. The HAMA includes 14 items, and a score >7 indicates possible anxiety. The HAMD includes 17 items, and a score >7 indicates possible depression. Sleep quality was evaluated using the Pittsburgh Sleep Quality Index (PSQI), which ranges from 0 to 21. A higher PSQI score indicates poorer sleep quality.

### 2.4. Statistical Analysis

All variables were analyzed using descriptive statistics. Continuous variables are presented as the mean and standard deviation (SD) or the median and range, and categorical variables are presented as the percentage. Two-tailed Student’s t-tests or Mann–Whitney U-tests were performed to compare continuous variables, and chi-square tests were used to compare categorical variables. The number of progressive stages and decreased ALSFRS-S scores were recorded at the 6-month follow-up. The sensitivity, specificity, and area under the curve of the receiver operating characteristic (ROC) curves of MITOS progression and ALSFRS-R scores decline (loss of one point or more) from baseline to 6 months on predicting the survival outcome at 12, 18, and 24 months were calculated and compared through Mann–Whitney U-tests. Predicted probabilities with the corresponding 95% confidence intervals (CI) of the MITOS progression from baseline to 6 months for survival outcome at 12, 18, and 24 months were calculated via the binary logistic regression models. Cox regression model was utilized to study the association between MITOS progression from baseline to 6 months and survival, and univariate hazard ratios (HRs) with the 95% CI were calculated. All statistical analyses were performed using SPSS 23.0 software. *p* values < 0.05 were considered statistically significant.

## 3. Results

### 3.1. Clinical Features and MITOS Progression in ALS Patients

**Figure 1:**
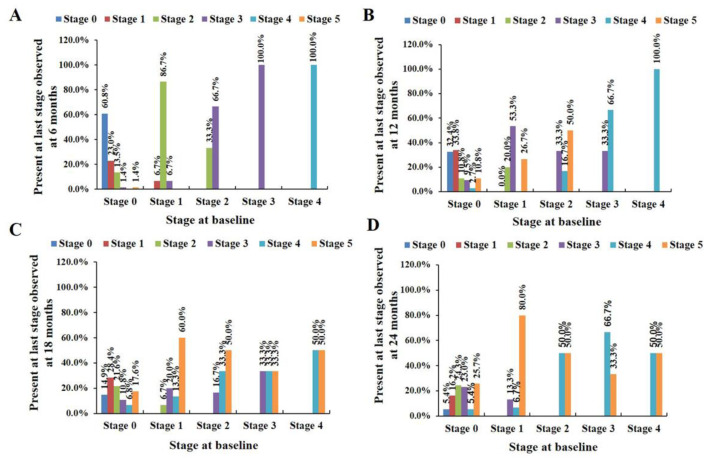
Stage progression of MITOS system at 6 months (**A**), at 12 months (**B**), at 18 months (**C**), and at 24 months (**D**) by baseline stage in ALS patients.

In total, 100 patients with ALS were included in this study. Seventy-four (74%) patients were male, and 32 (32%) patients had bulbar onset. The mean age at symptom onset was 52.58 ± 10.04 years, and mean age at diagnosis was 53.91 ± 10.22 years. The median delayed time to diagnosis was 12 months. The number of patients at each stage of the MITOS system and the number of those who progressed from one stage to another during follow-up are shown in Figure 1. At baseline, 74 patients were in stage 0 and 15 patients were in stage 1 based on the MITOS system, while only six patients were in stage 2, three in stage 3 and two in stage 4. Among the 15 patients in stage 1, 11 had lost function in walking or self-care, three in swallowing, and one in breathing. At the 6-, 12-, 18-, and 24-month follow-up visits, the percentages of patients who progressed one or multiple stages of the MITOS system were 47.0%, 72.0%, 87.0% and 95.0%, respectively. At 6-month follow-up, patients who were in stage 1 at baseline had a higher probability (87.5%) to progress to advanced stages than those in stage 0 (38.4%). At 24-month follow-up, 4 patients (4.0%) were in stage 0, 12 patients (12.0%) were in stage 1, 18 patients (18.0%) were in stage 2, 19 patients (19.0%) were in stage 3, 11 patients (11.0%) were in stage 4, and 36 patients (36.0%) were in stage 5. No patient moved backward to lower stages.

### 3.2. Comparison of MITOS Progression and ALSFRS-R Decline for Survival Outcome Prediction

Based on the MITOS system, irrespective of the stage at baseline, stage progression from baseline to 6 months indicated a sensitivity of 63.5% and a specificity of 100% for the prediction of the primary outcome at 12 months. At 18 months, MITOS progression from baseline to 6 months had the probability to predict the primary outcome with a sensitivity of 71.2% and a specificity of 92.6%. At 24 months, MITOS progression within the first 6 months from baseline could probably predict the primary outcome, with a sensitivity of 78.1% and a specificity of 88.9%, in a patient who progressed one or more stages (Table 1). A comparison of the predictive value of the primary outcome between MITOS progression and ALSFRS-R decline from baseline to the first 6 months was performed. As shown in Table 1, no significant differences were found between the ROC curve area of MITOS progression and that of ALSFRS-R score decline at 12-, 18-, and 24-month follow-up. Compared to ALSFRS-R decline, MITOS progression from baseline to 6 months showed comparative value for survival prediction at 12, 18, and 24 months, confirming the validity of the MITOS system.

### 3.3. Association between MITOS Progression during the Early Disease Course and Long-Term Survival in ALS Patients

**Figure 2:**
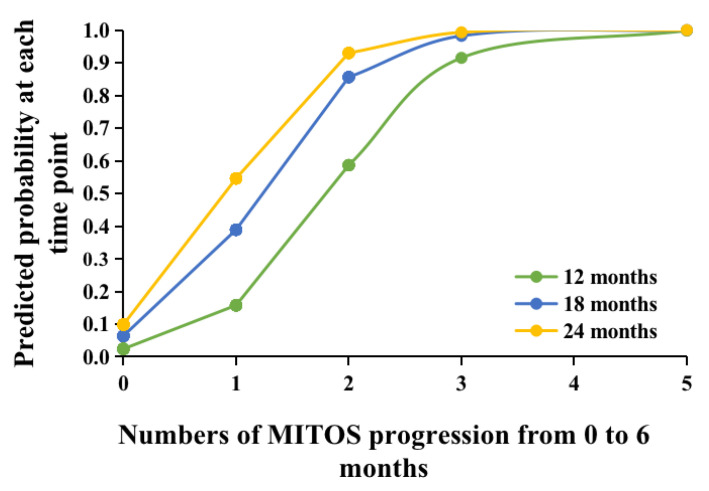
Predicted probability of numbers of MITOS system progression from baseline to 6 months follow-up for survival outcome at 12, 18, and 24 months follow-up.

**Figure 3:**
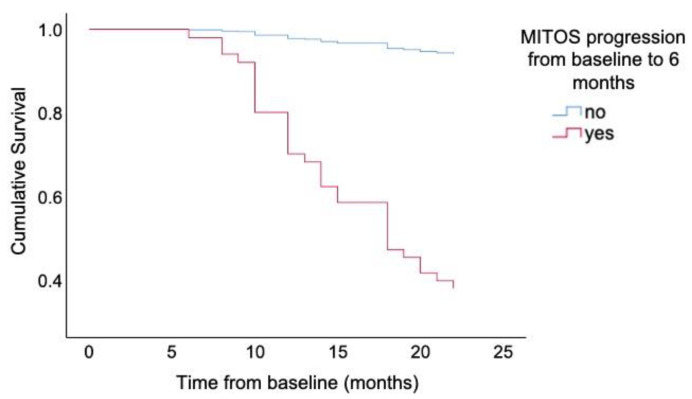
Survival curves of patients with MITOS progression and patients without MITOS progression from baseline to 6 months.

There was no relationship between factors at baseline, including age at diagnosis, sex, site of onset, duration before diagnosis, or neuropsychological scores, and the survival outcome at 12, 18, and 24 months through logistic regression analyses (data not shown). However, the number of patients who experienced progression according to the MITOS system from baseline to 6 months was an independent factor that was significantly associated with the occurrence of death at 12, 18, and 24 months. As MITOS progression increased by one stage within the first 6 months, the risk of death increased significantly. For instance, the risk of death at 24 months was 11.021 (95% CI 4.287–28.332, *p* < 0.001) times greater in ALS patients who experienced MITOS progression by three stages than in patients who experienced MITOS progression by two stages. The results of the logistic regression analyses of the MITOS system for predicting survival outcome in ALS patients are presented in Table 2. Furthermore, we found that the larger the number of MITOS progression, the higher the predicted probability of reaching death at any time point (i.e., patients who progressed by two stages from baseline to 6 months had a predicted probability of approximately 93% on the occurrence of death at 24 months, while the predicted probability was approximately 55% in patients who progressed by one stage from baseline to 6 months) (Figure 2). In addition, irrespective of the number of stages according to MITOS progression from baseline to 6 months, the predicted probability of death was highest at 24 months among all time points (Figure 2). Moreover, the univariate Cox regression analyses revealed that MITOS progression from baseline to 6-month follow-up was significantly associated with death (HR 15.956, 95% CI 4.843–52.573, *p* < 0.001). The survival curves of patients with MITOS progression and patients without MITOS progression from baseline to 6 months are shown in Figure 3. 

### 3.4. Factors Associated with MITOS Progression in ALS Patients

**Figure 4:**
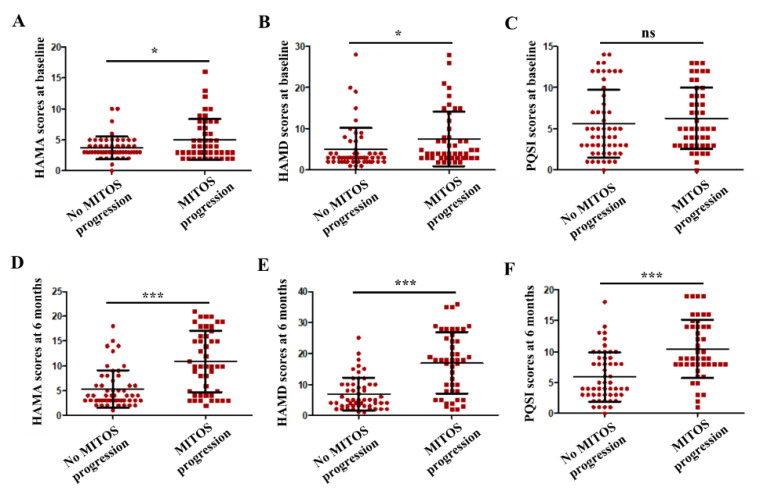
Comparison between patients with no MITOS progression and patients with MITOS progression on HAMA scores, HAMD scores, and PQSI scores at baseline (**A**–**C**), and HAMA scores, HAMD scores, and PQSI scores at 6 months follow-up (**D**–**F**). * *p* value < 0.05; *** *p* value < 0.001; ns: no significance.

Among all ALS patients, 47 (47.0%) had progressed by at least one stage from baseline to the 6-month follow-up based on the MITOS system. The demographics and clinical features of patients who did not experience MITOS progression and those who did in the first 6 months are presented in Table 3. Compared with those who did not experience MITOS progression, patients who did were significantly older at onset (*p* < 0.01) and at diagnosis (*p* < 0.01). There were no significant differences in the proportion of males or females, frequency of bulbar onset, delayed time to diagnosis, or ALSFRS-R scores between patients who did and did not experience MITOS progression from baseline to 6 months. In the group of patients who experienced MITOS progression, both the HAMA scores and HAMD scores at baseline were significantly higher (*p* < 0.05) than in those who did not experience MITOS progression, whereas the PQSI scores were not different between the two groups (Figure 4a–c). At the 6-month follow-up, compared with those who did not experience MITOS progression, patients who did experience MITOS progression from baseline to 6 months presented significantly higher HAMA scores, HAMD scores, and PQSI scores with all (*p* < 0.001) (Figure 4d,e).

## 4. Discussion

In the present longitudinal study, we confirmed the MITOS system to be consistent with the natural progression of ALS and validated the utility of the MITOS system in measuring disease burden in a Chinese cohort of ALS patients. In particular, our results revealed that MITOS progression from baseline to the first 6 months could reliably predict long-term survival (up to 24 months). Age at onset, age at diagnosis, and certain neuropsychological factors might be associated with MITOS progression during the early disease course.

ALSFRS-R decline has been employed to determine the rate of disease progression in several population-based studies [15,16] and as a primary or secondary outcome measure in most clinical trials on ALS [5,17,18]. However, the ALSFRS-R scale, which does not include milestones, is unable to detect functional changes in the later stages of ALS. By contrast, two main ALS clinical staging systems, the MITOS system and the King’s College staging system, include milestones and can be used to accurately evaluate the disease status and severity in ALS patients [8,9]. Compared with the King’s College staging system, the MITOS system has been demonstrated to be more sensitive to functional deterioration in the later disease stages and can serve as a complementary scale for ALSFRS-R [13,19]. Use of the King’s College staging system has been validated in a number of studies [9,11,20,21], whereas few reports have assessed the application of the MITOS system in ALS [8,10,22].

To our knowledge, the present study was the first longitudinal study to validate the MITOS system in a Chinese cohort of ALS patients. The findings of the current study revealed that age at onset and age at diagnosis were consistent with those reported in a large cohort of Chinese ALS patients [23,24,25], but earlier than those reported in a European cohort [1]. Approximately 1/3 of patients had bulbar onset, consistent with previous reports from the European population [1]. At entry, most ALS patients were in stage 0 of the MITOS system. As follow-up progressed, the proportion of patients who moved to advanced stages increased dramatically, and no patient was downstaged. Moreover, ALS patients who were in a higher Milano–Torino stage at baseline had a greater probability of progressing to a more advanced stage, indicating that severity during the early disease course affects the progression of ALS. The rate of MITOS progression from baseline to 6 months showed comparable sensitivity and specificity to that of ALSFRS-R decline in the first 6 months on survival prediction at 12, 18, and 24 months. Our results are consistent with those of a previous study and validate the use of the MITOS system in Chinese ALS patients [10].

ALS is a progressive neurodegenerative disease with prominent clinical and genetic heterogeneity. It has been demonstrated that certain clinical features, genetic factors, and therapeutic strategies can serve as prognostic biomarkers for survival in ALS patients. Clinically, old age at diagnosis, ALS with bulbar or respiratory onset and a poor nutritional status are likely associated with short survival [1]. In addition, previous studies have elucidated that ALSFRS-R decline is correlated with a short survival time [4,5,26]. In a recent study, Manera et al. reported that the regional spread of symptoms at diagnosis could be a prognostic marker in ALS [27]. Gaiani et al. demonstrated that neurofilament light chain in cerebrospinal fluid might have prognostic value in patients with ALS when combined with the MITOS system to measure disease progression [28]. In a study based on data from a clinical trial in Italy, Tramacere et al. revealed that the Milano–Torino stage at the 6-month follow-up could predict the survival outcome at 12 and 18 months [10]. However, whether the rate of MITOS progression during the early disease course of ALS is associated with long-term survival has not been evaluated before. We found that the rate of MITOS progression from baseline to 6 months was strongly associated with the survival outcome at 12, 18, and 24 months. In particular, with the increased number of MITOS progression from baseline to 6 months, the predictive probability of outcomes increased accordingly at any time point during the follow-up. Furthermore, the Cox regression analyses confirmed the significant association between MITOS progression at early disease course and death outcome.

Numerous studies have examined the potential risk factors affecting the survival of ALS patients, whereas factors influencing disease progression or functional deterioration have not been well elucidated. In a previous study, Yokoi et al. demonstrated that older age at onset was correlated with rapid progression of regional dysfunction and a short survival time in patients with sporadic ALS [29]. Another study showed that age at onset, female sex and initial symptoms (upper limb weakness, lower limb weakness or bulbar symptoms) were associated with early disease progression, but the factors affecting functional decline and survival outcome in ALS were different to some extent [26]. A recent study revealed that the electrophysiological spread pattern could affect the functional Milano–Torino stage in ALS patients [30]. Serological factors and cognitive impairment are also related to functional decline during disease progression in ALS patients [27,31]. In the present study, we found that patients who experienced MITOS progression were older at onset and diagnosis than those who did not experience MITOS progression from baseline to 6 months. Our findings suggest that age at onset and diagnosis might be associated with ALS progression, consistent with previous studies [26,29,32]. Apart from motor function disorders, cognitive impairment and neuropsychiatric symptoms are also common nonmotor manifestations in ALS patients [33,34]. In a previous study, emotional disorders such as depression and anxiety appeared to be the most common neuropsychiatric symptoms in Chinese ALS patients [35]. Sleep disturbances are also common in ALS patients and are probably associated with physical symptoms or mood disorders, including depression and anxiety [36,37]. It has been suggested that neuropsychiatric symptoms can be accompanied by motor function impairment or appear after the deterioration of movement disorders. The findings of the present study suggest that neuropsychiatric disorders such as depression and anxiety might be associated with MITOS progression during the early stages of ALS. The degrees of depression and anxiety appeared to be more severe in ALS patients who experienced MITOS progression from baseline to 6 months than in those who did not. Moreover, the aggravation of depression and anxiety, as well as MITOS progression, might have a joint impact on sleep quality. Further studies are required to elucidate the relationship among disease progression and these neuropsychiatric factors.

There are several limitations to the present study. First, the limited number of patients recruited in a single-center study might have led to a possible bias. Second, the clinical and neuropsychiatric features of patients in different Milano–Torino stages could not be carefully analyzed due to the small sample size. Moreover, a combination of the MITOS system and the King’s College staging system could be utilized to measure the disease progression of ALS in our future studies.

## 5. Conclusions

In conclusion, the findings of the present study suggest that the MITOS system can be used to evaluate disease progression in Chinese ALS patients. MITOS progression during the early disease course, which could serve as prognostic markers of long-term survival, may have utility in clinical trials. Age at onset and diagnosis and neuropsychiatric factors might be associated with disease progression measured via the MITOS system.

## Figures and Tables

**Table 1:** Comparison of MITOS progression and ALSFRS-R decline from baseline to 6 months on predicting survival outcome at 12, 18, and 24 months follow-up.

Time Point	Predicted Scale	Specificity	Sensitivity	ROC Curve Area (95% CI)	*p* Value
12 months	Numbers of MITOS progression	1.000	0.635	0.872 (0.790–0.931)	0.853
	Declined scores of ALSFRS-R	0.933	0.788	0.878 (0.797–0.935)	
18 months	Numbers of MITOS progression	0.712	0.926	0.857 (0.773–0.919)	0.969
	Declined scores of ALSFRS-R	0.863	0.815	0.858 (0.774–0.920)	
24 months	Numbers of MITOS progression	0.781	0.889	0.855 (0.771–0.918)	0.290
	Declined scores of ALSFRS-R	0.891	0.694	0.818 (0.728–0.888)	

MITOS: Milano–Torino Staging, ALSFRS-R: Amyotrophic Lateral Sclerosis Functional Rating Scale—Revised, ROC: Receiver Operating Characteristic, CI: Confidence interval.

**Table 2:** Odds ratios of MITOS progression from baseline to 6 months for predicting survival outcome in ALS patients via logistic regression analysis.

Variable	Time Point	OR (95% CI)	*p* Value
Numbers of MITOS progression from 0 to 6 months	12 months	7.584 (2.831–20.321)	<0.001
	18 months	9.342 (3.648–20.925)	<0.001
	24 months	11.021 (4.287–28.332)	<0.001

MITOS: Milano–Torino Staging, OR: Odds Ratio, CI: Confidence interval.

**Table 3:** Demographic and clinical characteristics of ALS patients without or with MITOS progression from baseline to 6 months.

Characteristic	Patients without MITOS Progression from 0 to 6 Months (n = 53)	Patients with MITOS Progression from 0 to 6 Months (n = 47)	*p* Value
Male, n (%)	39 (73.6%)	35 (74.5%)	0.920
Bulbar onset, n (%)	13 (24.5%)	19 (40.4%)	0.089
Age at onset (years)			
Mean (SD)	49.94 (10.00)	55.55 (10.13)	0.006 **
Median (range)	48 (32–78)	58 (33–73)	
Age at diagnosis (years)			
Mean (SD)	51.43 (9.80)	56.70 (10.06)	0.009 **
Median (range)	49 (33–79)	58 (33–74)	
Delay time to diagnosis (month)			
Mean (SD)	18.11 (14.36)	14.89 (12.49)	0.237
Median (range)	12 (3–62)	12 (2–60)	
ALSFRS-R score at baseline, Mean (SD)	40.28 (10.43)	38.34 (5.01)	0.248
HAMA score at baseline, Mean (SD)	3.72 (1.80)	5.06 (3.31)	0.012 *
HAMD score at baseline, Mean (SD)	4.98 (5.24)	7.57 (6.62)	0.031 *
PQSI score at baseline, Mean (SD)	5.62 (4.12)	6.28 (3.72)	0.409

MITOS: Milano–Torino Staging, ALSFRS-R: Amyotrophic Lateral Sclerosis Functional Rating Scale—Revised, HAMA: Hamilton Anxiety Scale, HAMD: Hamilton Depression Scale, PSQI: Pittsburgh Sleep Quality Index. ** *p* < 0.01; * *p* < 0.05.

## Data Availability

Not applicable.
